# Locating Underground Pipe Using Wideband Chaotic Ground Penetrating Radar

**DOI:** 10.3390/s19132913

**Published:** 2019-07-01

**Authors:** Jingxia Li, Tian Guo, Henry Leung, Hang Xu, Li Liu, Bingjie Wang, Yang Liu

**Affiliations:** 1 Key Laboratory of Advanced Transducers & Intelligent Control System, Ministry of Education and Shanxi Province, Taiyuan University of Technology, Taiyuan 030024, China; 2 College of Physics & Optoelectronics, Taiyuan University of Technology, Taiyuan 030024, China; 3 Department of Electrical and Computer Engineering, University of Calgary, University Drive, N.W., Calgary, AB 2500, Canada

**Keywords:** ground penetrating radar, chaotic signal, pipe location, plastic pipe location

## Abstract

An experimental wideband chaotic ground penetrating radar is proposed to locate underground pipes. A chaotic signal with a bandwidth of 1.56 GHz is utilized as the probe signal. The localization of the pipes is achieved by correlating the chaotic echo signal with its delayed duplicate and back-projection algorithm. Experimental results demonstrate that plastic pipe, metallic pipe, and multiple pipes can be located with a range resolution of 10 cm. Limited by the height of the sand, the detectable range is estimated to be 0.7 m for both the plastic pipes and the metallic pipes when the transmitting power is −12 dBm. The proposed system has the potential to detect buried pipes, and it is suitable for geological and civil engineering applications.

## 1. Introduction

Locating underground utility pipes is an essential task before excavation works. When underground pipes are damaged, they can lead to major financial losses and may cause loss of life. Various techniques have been developed for underground pipe detection, such as acoustic and vibration techniques [1,2], radio-frequency identification (RFID)/sensor techniques [3], infrared thermography [4], magnetic flux leakage method [5,6], eddy current technique [7,8], as well as ground penetrating radar (GPR) [9,10,11]. Among these non-destructive techniques, GPR is widely used because it can locate both non-metallic and metallic objects without prior knowledge.

There are different designs of GPR including impulse radar [12,13,14], frequency modulated continuous wave (FMCW) radar [15,16,17], stepped frequency continuous wave (SFCW) radar [18,19,20], and ultra wideband (UWB) signal radar [21,22]. Commercial GPR is mainly based on impulse signals. This technique has to compromise between the resolution and the detection distance. It is also limited by other signals, such as from plastic pipe or other non-metallic objects, that can be submerged in the background. FMCW and SFCW radars utilize large time-bandwidth signals to balance the resolution and detection distance. However, these two types of radar use the Inverse Discrete Fourier Transform to transform the data into the time domain, which can affect the high side lobe and thus harm plastic pipe detection. The Inverse Discrete Fourier Transform can be avoided by using inversion techniques in the frequency domain [23,24]. Furthermore, by applying inversion techniques in GPR, we can obtain a better reconstruction of buried targets, which is helpful for interpretation of the radargram [25,26]. In addition, this technique permits many applicative advantages such as high resolution imaging, and fast and effective data processing [27,28,29].

Nowadays, UWB has become a research hotpot in the field of radar, owing to its advantages of high range resolution and strong penetration. UWB radar transmits a wideband signal to the ground, and the received echo will contain information on the target with rich transitory response content. Here, we can see that an antenna is one of the essential components in this type of radar system. The antennas should be able to transmit and receive the ultra band spectrum with no distortion. Current studies show the great progress of UWB antennas [30,31,32]. UWB signal is another critical component. Traditional UWB radar utilizes short pulse as the transmit signals. But, a wide bandwidth introduces serious signal attenuation, and this will cause the echo signal from the target to be weak and even to be submerged in the background clutter and noise. In order to enhance the power of the echo signal, we have to increase the pulse repetition frequency or transmit an UWB-based modulation signal. However, the former can result in a decrease of the unambiguous detection distance. The latter can suffer from poor side lobe suppression characteristic, which decreases the effective range. Extensive studies have shown that UWB random signals can also be used as the transmit signals in UWB radar. UWB random radar utilizes noise signals [33] or pseudo random coded signals [34] as the transmit signal. It yields good depth resolution and has excellent anti-interference performance [35]. In addition, it possesses ideal “thumbtack” ambiguity function. All of these advantages are beneficial for weak signal detection. Narayanan et al. demonstrated the performance of the random noise radar and applied it to locate buried targets [36,37]. Tomizawa et al. proposed a random signal radar design based on M-sequence coded pulses [38], and the system can enhance the signal-to-noise ratio (SNR) and increase the detection range [39].

Chaotic signal is another type of random signal which has the following advantages: (1) compared with noise signals, a wideband chaotic signal with a large amplitude can be easily generated by simple nonlinear dynamical systems. The noise-like chaotic waveforms also have very broad bandwidths and good correlation properties that offer great range resolution and unambiguity in radar applications [40], (2) compared with pseudo random coded signals, a chaotic signal has no certain code length which will eliminate detection ambiguity. Therefore, chaotic signal has been widely applied to radar and has been verified to be a promising alternative [41,42,43,44,45,46,47].

In this paper, we propose to use wideband chaotic radar to locate underground pipe. The wideband chaotic signals are used for object location by the time domain correlation and back-projection (BP) algorithms. The main contribution of this paper is to introduce chaotic signals to pipe detection. Although pseudo random coded and noise signals have been applied to buried pipe detection, chaotic signals have been verified to have better correlation and more characteristics than pseudo random coded signals [48,49]. In addition, the SNR of the chaos-based radar can be improved by increasing the chaotic correlation length instead of amplifying the signal amplitude [50]. These properties can improve buried pipe detection, especially for plastic pipe detection. The paper is organized as follows: Section 2 provides the chaos radar system used in this study. Section 3 describes the characteristics of the detected signal. Section 4 shows the experimental results on pipe detection. Section 5 shows the comparison between stepped frequency signal radar and the proposed chaotic radars. Finally, conclusions are given in Section 6.

## 2. The Chaotic Radar System 

**Figure 1:**
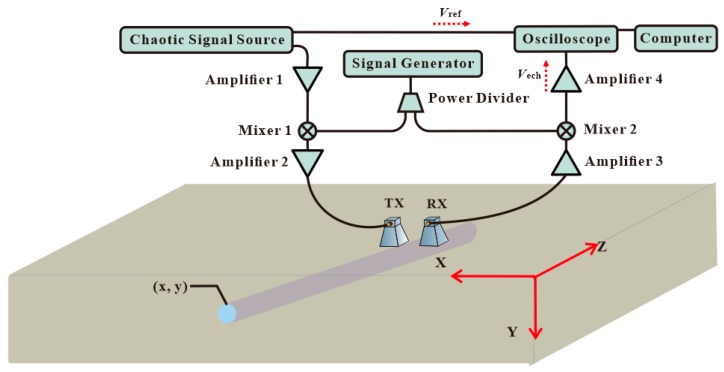
Block diagram of the chaotic radar system.

A block diagram of the chaotic radar system is shown in Figure 1. The wideband chaotic signal generated from a chaotic pulse position modulation (CPPM) circuit is split into two paths. One acts as the reference signal and is recorded by a real-time oscilloscope (RTO1024, ROHDE&SCHWARZ, Munich, Germany). The other is up-converted by a mixer 1 (M2-0026, Marki, Morgan Hill, CA, USA) via a 25 dB gain amplifier 1 (KG-RF-10, CONQUER, Beijing, China). It is then amplified by a 10 dB gain amplifier 2 (KG-RF-10, CONQUER,). The probe signal is transmitted by a wideband horn antenna (TX, LB-10180, A-INFO, Chengdu, China) with an operating frequency range of 1 GHz to 18 GHz and a reported gain of 11 dBi. The average power of the probe signal is −12 dBm. The echo signal from the buried pipe is received by a wideband horn antenna (RX), then it is amplified by a 20 dB gain amplifier 3 (KG-RF-10, CONQUER). After being down-converted by mixer 2 (M2-0026, Marki,) and amplified by a low noise amplifier 4 (11909A, Agilent, Santa Clara, CA, USA), the echo signal is recorded by the real-time oscilloscope. The signal generator (AV1487A, CETC, Qingdao, China) is used to provide the local oscillator frequency of mixers. The local oscillator frequency is 3.4 GHz. The outputs of the oscilloscope are transferred to a computer for data processing and display.

In the chaotic radar system, a wideband chaotic signal is used as a probe signal. Its auto-correlation is a delta-like function. Hence, depth information of the buried pipe can be obtained by correlating the echo signal and the reference signal. The correlation can be written as:

(1) $$V_{\mathrm{xcorr}}(t)=V_{\mathrm{ref}}(t)⊗V_{\mathrm{ech}}(t)=k\delta (t-\tau )$$

where ⊗ denotes the correlation operator, *V*_xcorr_(*t*), *V*_ref_(*t*), and *V*_ech_(*t*) are the correlation signal, reference signal, and the echo signal, respectively. *τ* represents the time delay between the echo signal and the reference, and *k* represents the system related constant. The depth of pipe can be obtained from the position of the correlation peak.

We utilize the BP algorithm after the correlation function to realize the two-dimensional (2D) imaging of the buried pipe. The TX and RX with a constant spacing move along the x-axis simultaneously from one end to the other on the sand surface. The whole imaging region is divided into *P* pixels. For any pixel point *p* in the imaging area, the signal *I_p_*(*t*) at this pixel is the sum of all envelopes of *V*_xcorr_(*t*) at the corresponding time delay. It can be expressed as:

(2) $$I_{p}(t)=\sum_{n=1}^{M}\left|V_{\mathrm{xcorr}}(t)\right|\delta (t-\frac{2R_{np}}{v})$$

where *R_np_* denotes the distance from pixel *p* to the *n*-th TX/RX, *M* is the unit of array elements, *v* is the velocity of the underground wave. If the pipe location is at pixel *p*, the energy at this position will be enhanced. Otherwise the energy will be small and can be regarded as background. The entire imaging area *I* can be written as:

(3) $$I=\sum_{p=1}^{P}I_{p}(t)$$

## 3. Characteristics of the Probe Signal

In our experiments, the wideband chaotic signal which is used as the probe signal is generated from a CPPM circuit [51]. The generated chaotic signal consists of pulse sequences with aperiodic intervals. This means that the pulse width is a constant, while the time interval between each pulse is chaotic and is produced by a logistic map [52].

**Figure 2:**
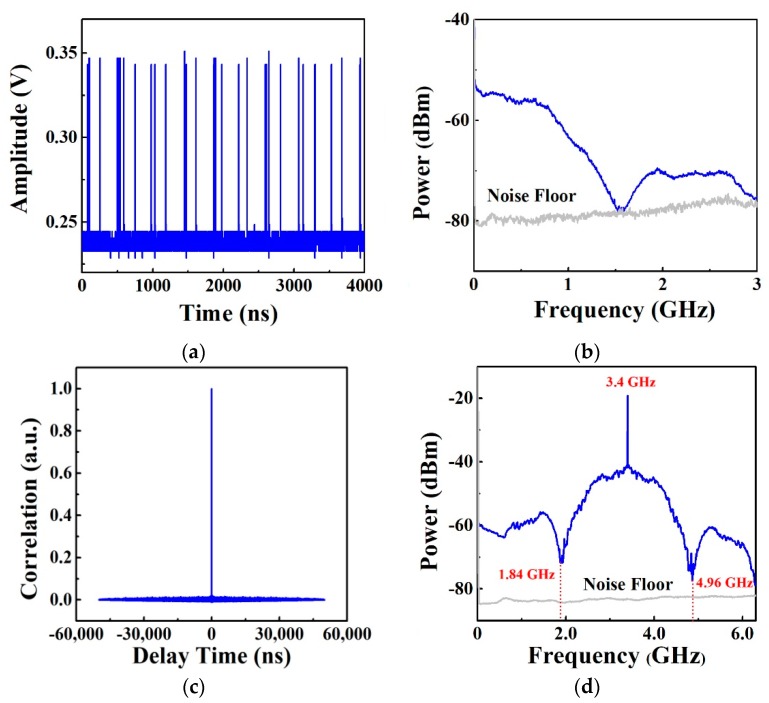
(**a**) Time series, (**b**) Power spectrum, and (**c**) auto-correlation trace of the probe signal, (**d**) power spectrum of the up-converted chaotic pulse position modulation (CPPM) signal as the transmitting signal.

Figure 2 shows the properties of chaotic signal. The time series of chaotic signal is shown in Figure 2a, and the amplitude of a single pulse is 0.1 V. From the power spectrum of the chaotic signal shown in Figure 2b, we see that the bandwidth is 1.56 GHz. The corresponding auto-correlation trace is depicted in Figure 2c. The power spectrum of the up-converted CPPM signal is used as the transmitting signal, as shown in Figure 2d. The CPPM signal with 1.56 GHz bandwidth is up-converted to 1.84–4.96 GHz through the mixer 1.

**Figure 3:**
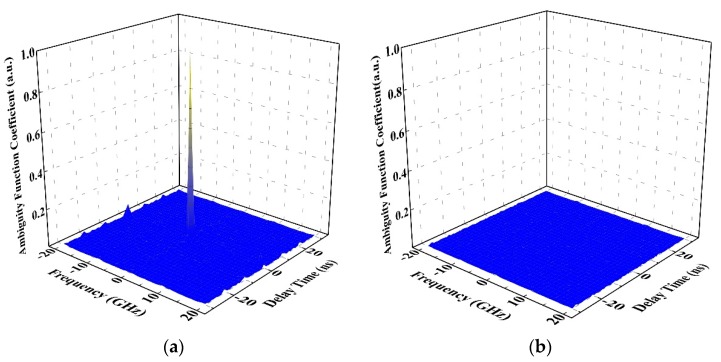
(**a**) An auto-ambiguity function and (**b**) cross-ambiguity function of the transmitted signal.

To demonstrate the performance of the wideband chaotic signal as the probe signal, the auto-ambiguity function and the cross-ambiguity function of the chaotic signal are provided in Figure 3a,b, respectively. The auto-ambiguity function has thumbtack peak with low sidelobes in both delay time and frequency axes. This illustrates that the chaotic signal used as the probe signal has excellent ability in unambiguous detection. From Figure 3b, we can see that there is no discernible spike on the surface, which indicates the chaotic radar has excellent electronic counter-countermeasure (ECCM) capability and anti-jamming property.

## 4. Experimental Results

**Figure 4:**
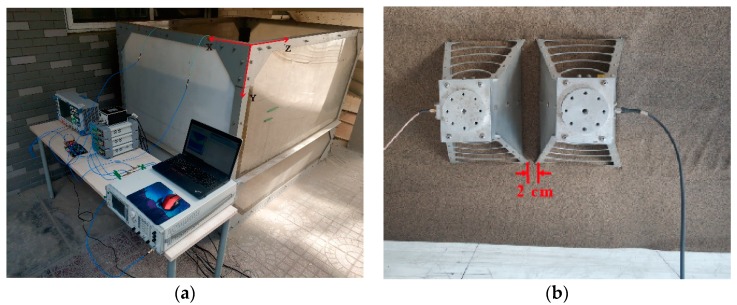
(**a**) The experimental scene of underground pipe location. (**b**) A photograph of the experimental tests in the sand container.

Figure 4a shows the experimental scene of pipe buried in dry sand. The tank is 2.00 m × 1.50 m × 1.50 m in size, and is made of acrylic with steel structure. In order to eliminate the strong interference of the steel, we have attached absorbing material to the surface of the steel. In the experiment, the antennas are placed on the surface of the sand, as shown in Figure 4b. They are moved along the x-axis with a step size of 0.05 m and the distance between the two antennas is 0.02 m. The parameters of the pipes, such as materials, diameters, lengths, and depths, are shown in Table 1. All of the pipes used in the experiment are filled with air. Limited by the measuring instrument, we cannot give the relative permittivity of the sand and the pipes. However, from the delay time and the known-depth of the pipe, we can deduce that the wave velocity is about 2 × 10^8^ m/s. Thus, the permittivity of the sand is about 2.25.

### 4.1. Plastic Pipe Location

**Figure 5:**
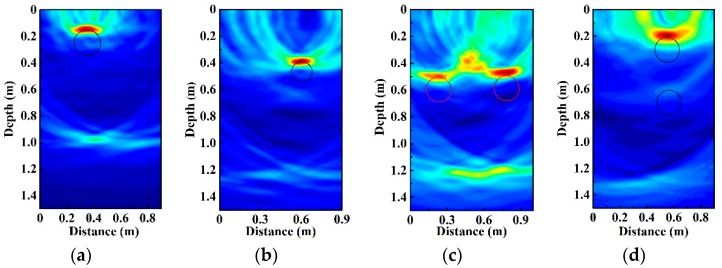
The radar profile of buried plastic pipes. (**a**) The position of plastic pipe 1 is at (0.35, 0.15) m. (**b**) The position of plastic pipe 2 is at (0.60, 0.38) m. (**c**) The positions of two plastic pipes 1 are at (0.22, 0.50) m and (0.76, 0.50) m. (**d**) The positions of two plastic pipes 1 are at (0.55, 0.20) m and (0.55, 0.60) m.

We first measure the plastic pipes of different diameter and buried depth. Plastic pipe 1 and plastic pipe 2 are buried 0.15 m and 0.38 m below the sand surface. The corresponding 2D images are shown in Figure 5a,b, respectively. They are obtained by using BP algorithm after correlating the chaotic echo signals with their delayed duplicates, which have been detailed in Section 2. Besides, average filtering is used to inhibit the direct wave reflected from the surface and crosstalk between TX and RX. In the figures, the position of signal trace corresponds to the top of the buried pipe and the circles represent the actual profile of the pipes. The results demonstrate that the depth of the plastic pipe can be obtained. In addition, the length of signal trace is related to the diameter of the pipe. The larger diameter of the pipe corresponds to the longer length of signal trace. Then we locate two plastic pipes buried in different positions. When the two pipes (Plastic 1) are at (0.22, 0.50) m and (0.76, 0.50) m, respectively, from Figure 5c we find that each pipe’s position can be identified clearly, and its center coordinate is close to the real value. When the two pipes (Plastic 1) are at (0.55, 0.20) m and (0.55, 0.60) m, respectively, we can locate both pipes, as shown in Figure 5d. Obviously, the signal trace of the upper pipe is clearer than the signal trace of the lower one. In Figure 5, we cannot observe multiple reflections of the plastic pipe, which may be related to the relative permittivity of the pipe. The reflection from the steel of the tank bottom can be seen. The above results verify that the chaotic GPR is capable of detecting buried plastic pipes.

### 4.2. Metallic Pipe Location

**Figure 6:**
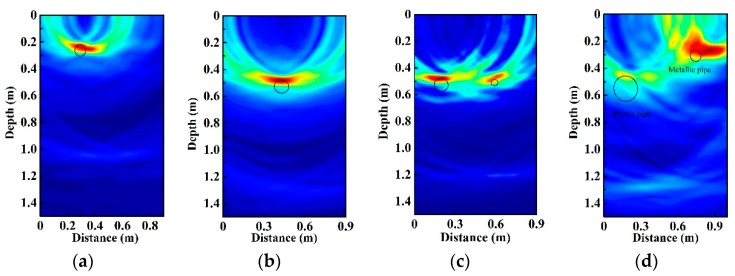
The radar profile of buried metallic pipes. (**a**) The position of metallic pipe 1 is at (0.30, 0.20) m. (**b**) The position of metallic pipe 1 is at (0.43, 0.45) m. (**c**) The positions of metallic pipe 1 and metallic pipe 2 are at (0.18, 0.50) m and (0.58, 0.50) m. (**d**) The radar profile of the two different type pipes.

Figure 6 demonstrates that the metallic pipe can be located. Metallic pipe 1 is buried at 0.20 m and 0.45 m below the sand surface. In Figure 6a,b, we can observe that the signal trace is clearly visible. Similarly, two different metallic pipes (metallic pipe 1 and metallic pipe 2) are buried at (0.18, 0.50) m and (0.58, 0.50) m, respectively. From Figure 6c, we can successfully detect both pipes. The larger diameter of pipe corresponds to a longer length of signal trace and this result is consistent with that of the plastic pipes. 

Furthermore, we try to locate two different material pipes simultaneously, which may give a better description of the real condition. Plastic pipe and metallic pipe are buried in sand from left to right, and their positions are (0.17, 0.45) m and (0.75, 0.25) m, respectively. Figure 6d indicates that the two different pipes can be located, and the metallic pipe shows stronger reflection because of the larger difference of dielectric properties between metal and sand. In addition, from Figure 6a–c, we cannot observe the reflection from the steel of the tank bottom since the signal is reflected back from the upper surface of the metal pipe. Our results demonstrate that the chaotic radar system can be applied to locate underground pipes.

### 4.3. Detection Performance

**Figure 7:**
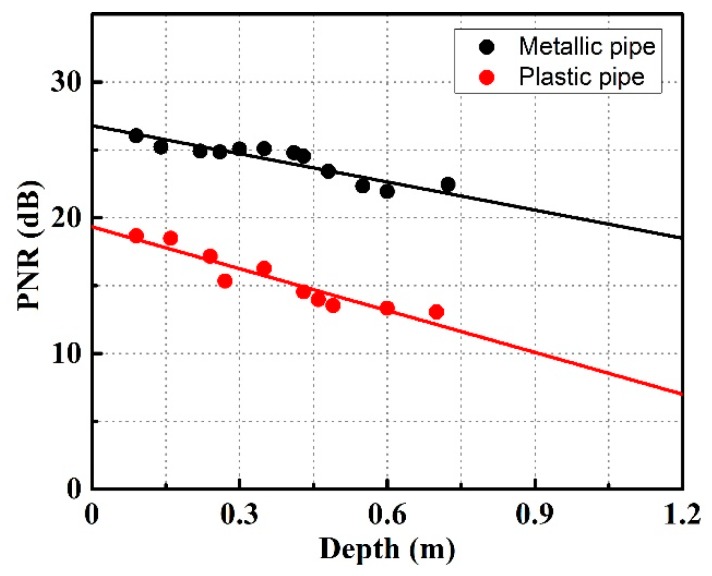
The PNRs of plastic pipe and metallic pipe as the function of buried depth.

We analyze the relationship between peak noise ratio (PNR) of the correlation curve and buried depth. The peak noise ratio (PNR) is defined as follows [53]:

(4) $$PNR=10\log_{10}\left(\frac{p}{\bar{n}+3\times std(n)}\right)$$

where *p* and *n* are the signal and noise value of the correlation trace, respectively. Figure 7 depicts the PNRs of the two type of pipes as the function of buried depth. From Figure 7, we observe that the PNR decreases with the increment of depth. Moreover, the detectable range of plastic pipe or metallic pipe is about 0.70 m. The PNR at the depth of 0.70 m is larger than 3 dB, which means deeper buried pipe can still be located by using this radar system. Unfortunately, as the sand slides easily, it is difficult to bury the pipe deeper. Besides, the height of sand is another limitation for range detecting.

**Figure 8:**
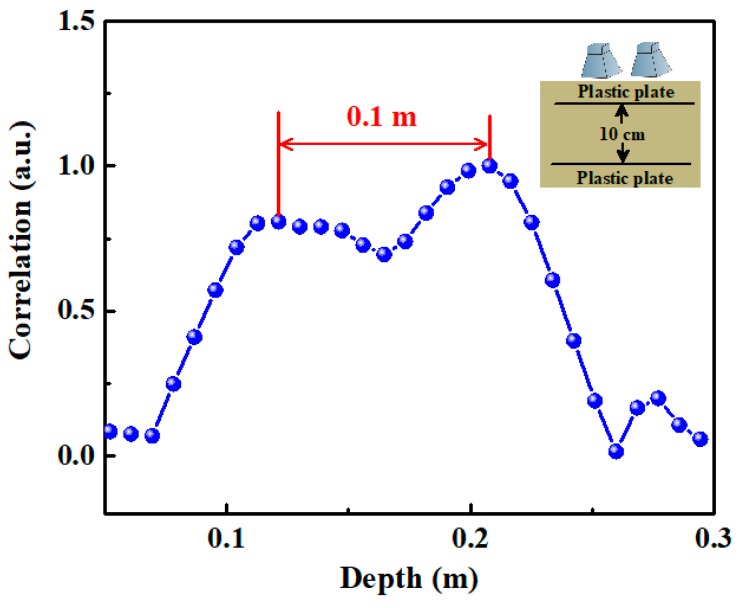
Identification of two reflection targets with 10 cm spacing.

To obtain the range resolution, we measure the position of two plastic plane reflecting targets, which are placed in the sand as shown in the inset. The sizes of the upper and lower plastic plates are 0.25 m × 0.40 m and 0.40 m × 0.60 m, respectively. They are about 1 mm thick. The lower plate is buried at 0.50 m below the antenna. Adjusting the depth of upper plate until their reflection peaks cannot be recognized from the correlation trace, we obtain a range resolution of 10 cm. Figure 8 shows the corresponding correlation trace.

## 5. Discussion

**Figure 9:**
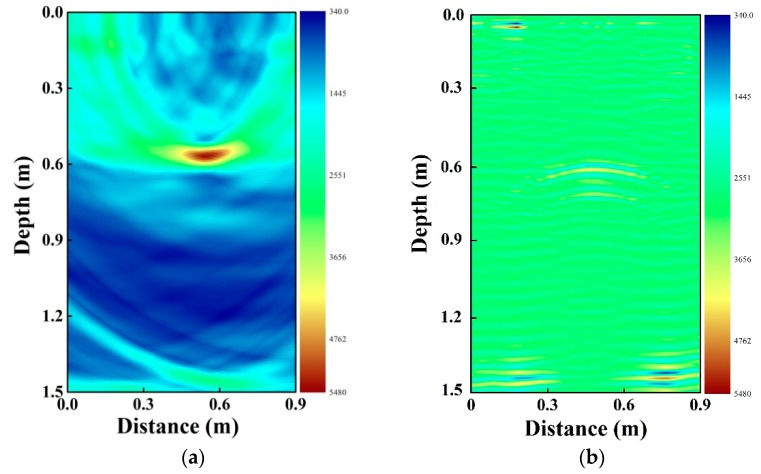
(**a**) Plastic location based on chaotic signals radar and (**b**) plastic location based on stepped frequency signal radar.

In order to analyze the performance of the proposed radar in underground pipe detection, we experimentally compare our chaotic radar with the stepped frequency signal radar in detecting plastic pipe. Here, the vector network analyzer (ZNB40, ROHDE&SCHWARZ, Munich, Germany) is regarded as the stepped frequency signal radar. Its modulated frequency range is from 1.8 GHz to 5.0 GHz, which covers the spectrum of the transmitting CPPM signal. Other experimental parameters in the two radars are the same, such as the transmitting power, the distance between the two antennas, the step of the two antennas, the pipe parameters, and their buried depth. Besides, average filtering is applied in the two systems. Figure 9 shows the compared result. Comparing the two figures, we can see that although plastic pipe buried at 0.60 m can be observed in the two figures, Figure 9a shows greater contrast between the pipe and the surroundings. This illustrates the value at the position of the pipe is larger than that of its surroundings. Thus, the proposed chaotic radar is capable of locating deeper pipe. We cannot compare the result of pipe location based on chaotic radar with pipe location based on other types of radars because of the limitation of the experimental conditions.

## 6. Conclusions

We propose and experimentally demonstrate a chaotic GPR for underground pipe location. The pipe is located by correlating the chaotic echo signal with chaotic delayed duplicate and BP algorithm. Experimental results demonstrate the chaotic GPR can realize underground pipe location. When the transmitting power is −12 dBm, the detectable range is about 0.7 m, which is limited by the height of the sand. The range resolution of the proposed radar system is 10 cm. The proposed chaotic GPR can achieve buried pipes’ location, and it is suitable for geological and civil engineering applications.

## Figures and Tables

**Table 1:** Summary of materials, diameters, lengths, and depths employed in the experiment.

Pipes	Aliasing	Diameter (m)	Length (m)	Thickness (mm)
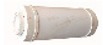	Plastic 1	0. 20	0.52	3
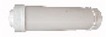	Plastic 2	0.15	0.60	4
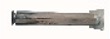	Metallic 1	0.10	0.60	2
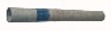	Metallic 2	0.05	0.35	1
